# 

**Published:** 1908-03

**Authors:** 


### BOOKS RECEIVED.

---

Surgery: Its Principles and Practice. In five volumes. By 66 eminent surgeons. Edited by W. W. Keen, M.D., Emeritus Professor of the Principles of Surgery and of Clinical Surgery, Jefferson Medical College, Philadelphia. Volume III. Octavo of 1132 pages, with 562 text-illustrations and 10 colored plates. Philadelphia and London: W. B. Saunders Company; 1908. (Per volume: cloth, $7.00; half morocco, $8.00, net prices.)

---

Syphilis. A Treatise for Practitioners. By Edward L. Keyes, Jr., A.B., M.D., Clinical Professor of Genitourinary Surgery in the New York Polyclinic Medical School and Hospital. Octavo, pp. 606. Illustrated. New York and London: D. Appleton and Company. 1908. (Cloth, $5.00.)

---

Practice of Medicine for Nurses. By George Howard Hoxie, M.D., Professor of Internal Medicine in the University of Kansas, Kansas City, Mo. 12 mo. pp. 284. Illustrated. Philadelphia and London: W. B. Saunders Company. 1908. (Cloth, $1.50.)

